# A study on the impact of role stress on engineer intention to leave in Indian construction firms

**DOI:** 10.1038/s41598-022-21730-2

**Published:** 2022-10-20

**Authors:** M. Hazeen Fathima, C. Umarani

**Affiliations:** Department of Civil Engineering, College of Engineering, Guindy, Anna University, Tamil Nadu, Chennai, 600025 India

**Keywords:** Psychology, Human behaviour

## Abstract

Construction jobs are stressful, and high employee turnover is one of the significant issues affecting the growth and development of construction firms worldwide. This study examines the relationship between role stress and intentions to leave, as well as the role of emotional exhaustion and job satisfaction as mediators in the role stress and intention to leave of engineers working in the construction sector. The sample of this study consists of three hundred and sixty engineers working in private construction firms across southern parts of India. The relationship between the study variables is examined using structural equation modeling. The result of the study reveals a significant positive relationship between role conflict and intention to leave, whereas the direct relationship between role ambiguity, role overload, and intention to leave is not significant. In terms of mediation, emotional exhaustion plays a significant mediator between all the components of role stress and intention to leave. At the same time, the role of job satisfaction is significant only between role ambiguity and intention to leave. Role ambiguity negatively influences job satisfaction, subsequently increasing engineers’ turnover intention. Emotional exhaustion caused due to role stress is identified as one of the significant predictors of engineers’ intention to leave. Thus, construction organizations should focus on redesigning construction jobs to reduce role stress and emotional exhaustion to retain engineers working with them.

## Introduction

Human capital is considered one of the valuable assets essential for the sustainable growth and development of any construction organization. The professionals' skills and efficiency will decide the construction project's quality and success^1,2^. High employee turnover is a primary concern affecting the construction sector^3^. Turnover of construction professionals is found to have been associated with delays in the project, increased project costs, poor project quality, and loss of expertise of the departing personnel, all of which have an impact on a construction firm's productivity, performance, and competitive advantage^3,4^. Employee turnover is harmful to any firm and psychologically impacts the organization and the employees that stay. Besides, additional costs and time losses are incurred due to the recruitment and training of new staff^3,4^. Furthermore, there is also a severe scarcity of skilled professionals in the construction sector globally^5^. Hence, studies focusing on employee turnover are essential to help construction firms formulate strategies to reduce employee turnover.

Employees' intent to leave the company is referred to as turnover intention, which is one of the best predictors of actual turnover^6^. Stress associated with the nature of the job has been identified as one of the critical antecedents of employee turnover in the construction sector^4,7^. Construction jobs are complex, dynamic, risky, and stressful. Planning, execution, and completion of construction projects must be done at high quality with limited time, budget, and resources^8,9^. Construction professionals, especially engineers, are exposed to several job-related challenges, as they play a significant role in construction projects' planning, execution, and completion. Role stress is one of the critical challenges faced by professionals working in the construction sector^7^. Heavy workloads, long working hours, ever-changing work demands, and stringent deadlines make their job roles more stressful compared to other industries^10^. Role stress is associated with reduced job satisfaction, poor performance, emotional exhaustion, and a strong desire to leave^11,12^.

The effect of role stress on employee intention to leave or turnover intention has been studied across several sectors, and it has been found that role stress, including role ambiguity, role conflict, and role overload, significantly influences employee turnover intention^11,13^. Although studies examining the relationship between role stress and employee turnover intention have been conducted in the construction sector, studies focusing on the mechanisms and processes that mediate the relationship between role stress and employee turnover are very few^7^. Especially in countries like India, where a high turnover of engineers and increasing work stress have been widely reported^14,15^, it is essential to improve the understanding regarding the degree of impact the different components of role stress have on the construction professionals' decision to leave. Hence, our study aims to examine the relationship between role stress (RA, RC & RO) and turnover intention and also investigates the role of emotional exhaustion and job satisfaction in the relationship between role stress and engineers' intention to leave the Indian construction sector. The insights obtained from this study would help formulate strategies for reducing the turnover of professionals, especially engineers in the construction sector.

## Theoretical background and hypothesis development

### Role stress and intention to leave

Role stress refers to the stress associated with one's job. Role stress occurs when there is an inability in individuals to learn and understand their rights and responsibilities at work^16^. Previous research has failed to reach a strong consensus on the dimension of role stress. Some offer a two-dimensional framework for role stress that includes role ambiguity (RA) and role conflict (RC)^11^, whilst others propose a three-dimensional structure that includes role overload (RO) as an additional dimension^13^.

Role ambiguity refers to one's belief that the job descriptions are unclear, causing them to be unsure where to focus their efforts. At the same time, conflicting expectations imposed on individuals in their jobs are referred to as role conflict. Role conflict occurs when an employee's job demands are inconsistent or incompatible^17^. Role overload, on the other hand, refers to an employee's perception that their job requirements are excessive compared to their available capabilities and resources^18^. Due to the dynamic and stressful nature of construction jobs, engineers are exposed to role stress^7,8^. They have to deal with complex job requirements, time constraints, work-related uncertainties, and work overload in addition to designing, planning, and executing construction projects^9,10^. Engineers working on construction projects may perceive role ambiguity when their job descriptions are unclear, and they do not have adequate information to complete their work tasks^7^. Likewise, due to the involvement of multiple parties in construction projects, they receive incompatible demands from multiple parties, which often results in role conflict^19^. Moreover, due to the advancement in construction processes and the increase in the need for skilled professionals, engineers working on construction projects are often subjected to work overload^7,20^.

Role stress up to a tolerable limit can result in beneficial work outcomes, such as enhanced motivation and performance^21^. If it surpasses the tolerable limit, it will cause negative behavior and energy depletion^22^. Role stress reduces employees' job involvement, increasing their psychological withdrawal and turnover intention^13,23^. Turnover intention or intentions to leave is the last stage of withdrawal cognitions, which ranges from thinking about leaving to wanting to look for an alternative job^6^. According to role theory, when the expectations in the role are unclear and inconsistent, employees feel stressed, which leads to discontent and turnover intention^17^.

Construction jobs are considered more stressful than other sectoral jobs^8^. With the prevalence of high employee turnover and skill shortage issues across construction organizations, it is essential to examine the degree of impact different components of role stress have on employees' intention to leave the construction sector. Role stress has been identified as one of the significant predictors of employee intention to leave the construction sector^7^. Previous works of literature conducted in other sectors also affirm the significant positive association between role stress and turnover intention^11,13,24^. Thus, the following hypothesis is proposed:

#### H1

- Role ambiguity is positively associated with employees' intention to leave.
- Role conflict is positively associated with employees' intention to leave
- Role overload is positively associated with employees' intention to leave

### Emotional exhaustion as a mediator between role stress and intention to leave

Emotional exhaustion, one of the core dimensions of burnout, indicates feeling emotionally exhausted and drained. It occurs when the job demand exceeds employees' capacity to work^25^. Highly demanding and stressful jobs have been recognized as potential causes of emotional exhaustion^26^. Employee emotional exhaustion will lead to poor commitment, job dissatisfaction, lower performance, and increased intention to quit^24,27–29^. Role stress components such as role ambiguity, role conflict, and role overload are positively associated with employees’ emotional exhaustion^24,30–33^. Emotional exhaustion of employees is found to increase with an increase in role stress. When employees are exhausted, they may connect their dissatisfaction to a disconnect with the organization. When they believe their condition cannot be improved and they do not have access to resources to help them, their perspectives and behaviors may suffer, and they may consider quitting their job. The conservation of resources theory best explains the impact of emotional exhaustion on turnover intention^34^. According to this theory, role stress is considered a threat to an individual's resources. When individuals suffer some losses (emotional exhaustion) because of such threats, they withdraw from their job as a coping mechanism to prevent further resource loss^34^.

The construction sector is considered one of the most stressful industries, and emotional exhaustion occurs more commonly among construction professionals because of their highly demanding work nature^12,35^. Mitigating employees' emotional exhaustion has become a major concern among construction organizations because of its positive association with employee intention to leave^12,35,36^. When engineers are subjected to role stress and lack the means to alleviate it, they may leave their job as a coping mechanism^12,35^. Hence a better understanding of the relationship between role stress, emotional exhaustion, and employees' intention to leave the construction sector is essential to formulate strategies to reduce emotional exhaustion, which in turn helps construction organizations in reducing employee turnover and the cost associated with it.

Previous studies have found a significant positive association between emotional exhaustion and employees' intention to leave^12,24,35,36^. Furthermore, the relationship between role stress and employees' intention to leave is mediated through emotional exhaustion, indicating that an increase in role stress increases emotional exhaustion among employees, which subsequently increases their intention to leave^13,23,24,37^. Based on these considerations, the following two hypotheses are proposed:

#### H2

Emotional exhaustion is positively associated with employees' intention to leave.

#### H3

- Emotional exhaustion mediates the impact of role ambiguity on employees' intention to leave
- Emotional exhaustion mediates the impact of role conflict on employees' intention to leave.
- Emotional exhaustion mediates the impact of role overload on employees' intention to leave

### Job satisfaction as a mediator between role stress and intention to leave

Job satisfaction is the measure of the employees' degree of satisfaction and happiness with their job^38^. It is also perceived as the emotional state resulting from comparing actual job outcomes with those desired^39^. Employees’ behavior in job and work outcomes is found to have to vary based on their job satisfaction. Employee job satisfaction is positively related to employee performance, productivity, and retention. Conversely, decreased job satisfaction will lead to emotional exhaustion, absenteeism, and employee turnover^6,39^.

Employee job satisfaction significantly predicts turnover intention in the construction sector^3,40^. Employees who are satisfied with their job stay longer^41^, whereas those who are dissatisfied leave their job early^3,7^. Several studies have examined the relationship between job satisfaction and employees' intention to leave the construction sector. For example^3^, studied the turnover intention of project managers in China, and his study concluded that an increase in project managers' overall job satisfaction leads to a decrease in their intention to leave. Likewise, the study conducted by^7^ among professionals in the Srilankan construction industry also revealed similar results. It is inferred from the above findings that an increase in construction employees' overall satisfaction with their job reduces their turnover intention. Hence construction organizations should focus on improving the overall job satisfaction of their employees to reduce their turnover.

**Figure 1:**
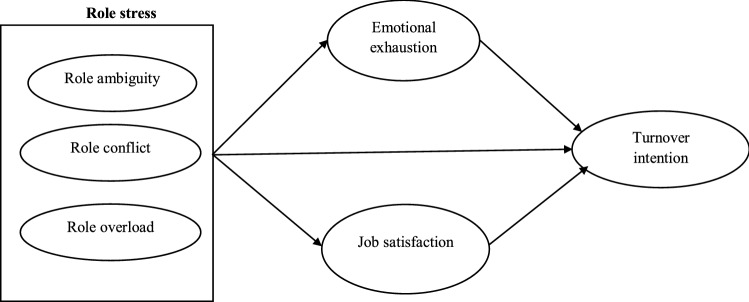
Conceptual framework of the study.

Previous research suggests a negative relationship between role stress (RA, RC, and RO) and employees' job satisfaction^42–44^. An increase in role stress decreases employees’ job satisfaction, increasing their intention to quit^7^. Based on that, work satisfaction may be seen as a buffer between role stress and the desire to leave. The mediation effect of job satisfaction between role stress and intention to leave has also been supported empirically by previous studies conducted across several sectors^7,43,45^. Based on that, the following hypothesis is proposed. Figure 1 illustrates the hypothesized conceptual framework.

#### H4

Job satisfaction is negatively associated with turnover intention.

#### H5

- Job satisfaction mediates the impact of role ambiguity on employees' turnover intention
- Job satisfaction mediates the impact of role conflict on employees' turnover intention.
- Job satisfaction mediates the impact of role overload on employees' turnover intention.

## Methodology

### Sample

The data were collected from the 360 engineers working on construction projects across southern parts of India using a questionnaire survey. The respondents were selected and contacted through alumni associations and professional networks. Both hard copy format and questionnaire link of google forms were used for data collection. The questionnaire survey was conducted for six months, from November 2019 to April 2020. This study was approved by the institutional review board of Anna University. All methods were performed in accordance with the relevant guidelines and regulations. Informed consent was obtained from all participants before completing the questionnaire. All the participants were informed about the nature and purpose of the research, and it was assured that their responses would be kept anonymous and used only for research purposes. A cover letter stating the researchers' affiliation and assurances of anonymity and confidentiality was provided along with the questionnaire. A total of 314 electronic surveys and 46 paper questionnaires were returned. Overall, 360 fully completed replies were gathered from 450 distributed surveys, reflecting an 80% response rate. The sample size of this study is considered appropriate because the final sample size of 360 included in this study exceeds the minimum sample size requirement value (S) of 271 obtained using the Cochran sample measure formula^46^. The Cochrans formula for an unknown population used for the calculation is given in Eq. 1. The minimum sample size requirement was calculated for an unknown population at a 90% confidence interval (Z), 5% margin error (e), and an assumed population proportion (*p*) value of 0.5.

1 $$\frac{{{\text{S}} = {\text{Z}}^{{2}} {\text{P}}\left( {{1} - {\text{P}}} \right)}}{{e^{2} }}.$$

### Participants and procedure

The participants of this study were instructed to complete a questionnaire either online or by hand with a pen or pencil. The survey consists of three parts. The first part explained the objective of the research study. The second part collects demographic information from respondents, such as gender, age, tenure, experience, and educational qualification. In the third part, employees' perceptions regarding study constructs such as role stress, emotional exhaustion, job satisfaction, and intention to leave were measured using a five-point scale ranging from 1 to 5 (strongly disagree to strongly agree). The construct items were shuffled randomly to avoid common method bias. The questionnaire was refined based on a pilot study conducted among 50 engineers. Some items were removed based on factor loading (factor loading < 0.3) and reliability values (alpha value < 0.7). The Cronbach alpha value for all the scales used was found to be greater than 0.7, which is acceptable^47^. The questionnaire items used in the study are presented in Table 1.

**Table 1:** Measures used in the study.

Variable name	Scale items
Role stress	
Role ambiguity	I feel certain about how much authority I have (R)
	Clear, planned goals and objectives exist for my job (R)
	I know that I have divided my time properly (R)
	I know what my responsibilities are (R)
	I know exactly what is expected of me (R)
	Explanation is clear of what has to be done (R)
Role conflict	I have to do things that should be done differently
	I receive an assignment without the manpower to complete it
	I have to buck a rule or policy in order to carry out an assignment
	I work with two or more groups who operate quite differently
	I receive incompatible requests from two or more people
	I do things that are apt to be accepted by one person and not accepted by others
	I receive an assignment without adequate resources and materials to execute it
	I work on unnecessary things
Role overload	“It often seems like I have too much work for one person to do
	The amount of work I am expected to do is too great.”
	I never seem to have enough time to get everything done at work
Emotional exhaustion	I feel emotionally drained due to my Job
	I feel fatigue when I get up in the morning as I have to face another day on the job
	I feel I'm working too hard on my job
	I feel frustrated by my job
Job satisfaction	I find real enjoyment in my job
	I like my job better than the average person
	Most days I am enthusiastic about my job
	Overall, I feel well satisfied with my current job
Intention to leave	I often think of leaving the organization
	I intend to look for a new job within the next year
	If I could choose again, I would not work for this organization

### Measure

- *Role stress*: Employees' perception regarding role stress was assessed through a 3 component scale consisting of role ambiguity, role conflict, and role overload. The six-item role ambiguity scale and the eight-item role conflict scale were developed based on^17^, whereas the three-item role overload scale was developed based on^48^. All the items in the scales were rated on a five-point Likert scale ranging from 1 = “strongly disagree” to 5 = “strongly agree”. The Cronbach’s alpha for role stress components ranged from 0.80 to 0.88.
- *Emotional exhaustion*: Employees' emotional exhaustion was measured using a four-item emotional exhaustion scale developed based on^49^. A five-point Likert scale ranging from 1 = “strongly disagree,” and 5 = “strongly agree” has been used to rate the scale items. The Cronbach’s alpha obtained for the emotional exhaustion scale used in this study was found to be 0.86.
- *Job satisfaction*: Employees' overall job satisfaction with their job was measured with a four-item scale developed based on^50^. The Cronbach's alpha value for the job satisfaction scale used is 0.75. The respondents were asked to rate their satisfaction on a five-point Likert scale ranging from 1 = “very dissatisfied” and 5 = “very satisfied
- *Turnover intention*: The turnover intention was measured using a three-item measurement^51^. The items were rated on a five-point Likert scale, where 1 = “strongly disagree” to 5 = “strongly agree”. The Cronbachs’ alpha value for the turnover intention scale used was 0.78.
- *Control Variable:* Gender, age, education levels, and work tenure were used as control variables in this study

### Data analysis

Data analysis was done using AMOS 24.0. Confirmatory factor analysis (CFA) was used to assess the scales' validity, and structural equation modeling was used to test the link between variables in the study model. The maximum likelihood estimation was used to determine the model fit.

## Results

### Demographic details

Out of the 360 respondents, 85.3% were male. The respondents were engineers, and most of them (refer to Table 2) were undergraduates (45.6%), between 21 and 30 years of age (81.7%), and had been working in the organization with experience of up to three years (51.4%).

**Table 2:** Demographic details of respondents.

Demographic characteristics	Frequency	Percentage
Sample size	360	
**Gender**		
Male	307	85.3
Female	53	14.7
**Age (years)**		
21–30	294	81.7
31–40	54	15
41–50	9	2.5
51–60	2	0.6
> 60	1	0.3
**Educational qualification**		
Post graduate	123	34.2
Under graduate	164	45.6
Diploma	73	20.3
**Experience(years)**		
0–3 years	185	51.4
3–6 years	87	24.2
6–9 years	48	13.3
Above 9 years	40	11.1

### Descriptive statistics

Table 3 shows the descriptive statistics and correlation values of the study variables. Role ambiguity (RA) is positively associated to turnover intention or intention to leave (TI) (r = 0.347, *p* < 0.01). Role conflict (RC) is positively associated to intention to leave (r = 0.402, *p* < 0.01), and role overload (RO) is also positively associated to intention to leave (r = 0.227, *p* < 0.01). Likewise, emotional exhaustion (EE) is positively associated with intention to leave (r = 0.557, *p* < 0.01) and job satisfaction (JS) is negatively associated with intention to leave (r =  − 0.180, *p* < 0.01). The results of the bivariate Pearson correlation provide initial support to the proposed hypothesized model.

**Table 3:** Results of descriptive statistics and correlations. Source: Made by the authors.

Variables	Mean	SD	1	2	3	4	5	6
1.RA	2.61	1.27	1.00					
2.RC	3.29	1.16	0.481**	1.00				
3.RO	3.37	1.15	0.296**	0.315**	1.00			
4.EE	3.18	1.23	0.499**	0.462**	0.361**	1.00		
5.JS	3.52	1.11	− 0.160**	− 0.154**	− 0.040	− 0.163**	1.00	
6.TI	3.34	1.29	0.347**	0.402**	0.227**	0.557**	− 0.180**	1.00

*RA* Role ambiguity, *RC* Role conflict, *RO* Role overload, *EE* Emotional exhaustion, *JS* Job satisfaction, *TI* Turnover intention, *SD* Standard deviation.

### Confirmatory factor analysis (CFA)

The final measurement model was tested with CFA. The loadings of each factor were found to be statistically significant (*p* < 0.001), with loadings 0.71–0.85 greater than the suggested value of 0.5^52^. The chi-square test, normed fit index (NFI), goodness-of-fit index (GFI), comparative-fit index (CFI), tucker-lewis coefficient index (TLI), and root-mean-square error of approximation (RMSEA) were all examined. The results revealed an acceptable fit χ2 = 206.48, χ2/df = 1.50, CFI = 0.980, TLI = 0.975, GFI = 0.951, NFI = 0.944, and RMSEA = 0.035, as the values obtained were within the acceptable range (χ2/df < 3, CFI > 0.9, TLI > 0.9, GFI > 0.9, NFI > 0.9, and RMSEA < 0.08)^53^.

### Discriminant and convergent validity

The study variables (RA, RC, RO, Emotional exhaustion, Job Satisfaction, and intention to leave) are distinct from one another since the correlation values between the constructs are smaller than the square roots of the average variances extracted (AVE) (Refer to Table 4). Thus, discriminant validity has been established. To test the convergent validity of all constructs, composite reliability (CR) and average variance extracted (AVE) values were obtained. The components' convergent validity is simultaneously validated by composite reliability scores ranging from 0.820 to 0.846 (above 0.6) and average variance extracted scores ranging from 0.595 to 0.648 (above 0.5)^47,52^. Harman's one-factor test was used to determine the common method variance (CMV). The variation in the data was only 29%; hence common method variance was not a major problem.

**Table 4:** Result of validity analysis. Source: Made by the authors.

Variables	CR	AVE	SIC					
			RA	RC	RO	EE	JS	TI
RA	0.820	0.620		0.306	0.085	0.277	0.036	0.169
RC	0.832	0.595	0.306		0.103	0.253	0.008	0.215
RO	0.827	0.633	0.085	0.103		0.140	0.016	0.045
EE	0.846	0.604	0.277	0.253	0.140		0.011	0.293
JS	0.819	0.648	0.036	0.008	0.016	0.011		0.093
TI	0.833	0.621	0.169	0.215	0.045	0.293	0.093	

*RA* Role ambiguity, *RC* Role conflict, *RO* Role overload, *EE* Emotional exhaustion, *JS* Job satisfaction, *TI* Turnover intention, *CR* composite reliability, *AVE* Average variance extracted, *SIC* squared inner construct correlation estimates.

### Results of hypotheses testing

**Figure 2:**
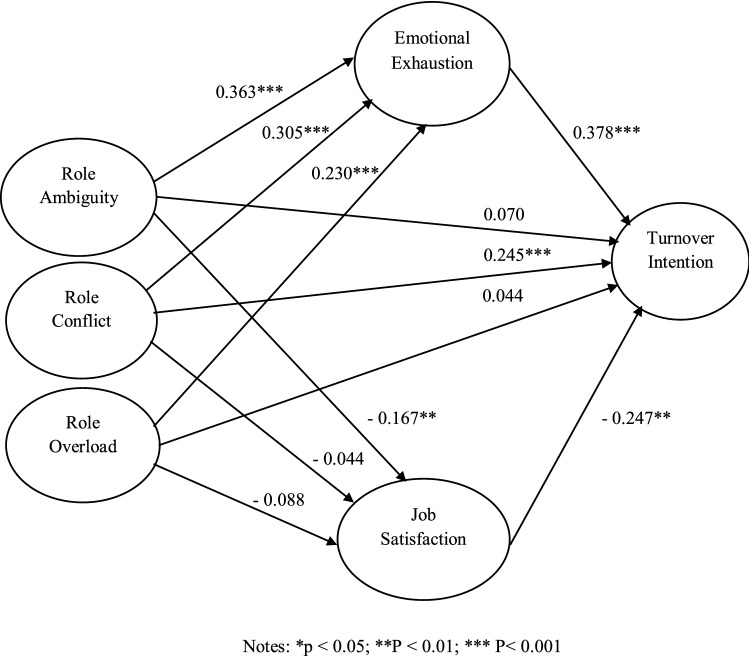
Structural model with standardized path coefficients.

After the confirmatory factor analysis, the structural model was used to test the conceptual framework and the proposed hypothesis. The proposed structural model shows an acceptable fit (χ2/df = 2.099, CFI = 0.950, TLI = 0.939, GFI = 0.951, NFI = 0.909, and RMSEA = 0.055)^54^. Among the direct path between role stress and turnover intention, only the direct path of RC to intention to leave was significant (β = 0.245, *p* < 0.001, H1 (b) supported). The direct path coefficient of RA to intention to leave (β = 0.070, p > 0.05, H1 (a) not supported) and RO to turnover intention (β = 0.044, *p* > 0.05, H1 (c) not supported) indicate no direct relationship (Refer Fig. 2). Hence the results partially support hypothesis H1. Besides that, the positive relationship between emotional exhaustion and turnover intention (β = 0.378, *p* < 0.001), as well as the negative relationship between job satisfaction and turnover intention (β =  − 0.247, *p* < 0.001), are also significant, supporting hypotheses H2 and hypothesis H4.

The test of mediating variables in the third and fifth hypotheses was done using the bootstrap method^55^. Two thousand bootstrap samples and a 95% confidence interval are used. The mediating role of emotional exhaustion in the role ambiguity (RA) and turnover intention (*p* < 0.001, H3 (a) supported), role conflict and turnover intention (*p* < 0.01, H3 (b) supported), and role overload and turnover intention relationship (*p* < 0.001, H3 (c) supported) are significant and the 95% confidence interval does not include zero supporting Hypothesis H3. The mediating effect of job satisfaction in the role stress turnover intention relationship is not fully confirmed. The mediating effect of job satisfaction is significant only in the role ambiguity (RA) and turnover intention relationship (*p* < 0.05, H5 (a) supported), and it is not significant in the role conflict (RC) turnover intention relationship (*p* > 0.05, H5 (b) not supported) and role overload (RO) turnover intention relationship (*p* > 0.05, H5 (c) not supported), partially supporting hypothesis H5 (Refer Table 5).

**Table 5:** Results of bootstrapping analysis.

Hypothesis	Estimated effect (β)	95% confidence interval			*p*-value	Results
		Lower bound confidence interval	Upper bound confidence interval			
RA – EE – TI	0.108	0.058	0.198		0.000	Supported
RC – EE – TI	0.111	0.052	0.206		0.001	Supported
RO – EE – TI	0.090	0.035	0.182		0.000	Supported
RA – JS – TI	0.032	0.004	0.074		0.022	Supported
RC – JS – TI	0.001	− 0.043	0.032		0.962	Not supported
RO – JS – TI	0.023	− 0.012	0.077		0.183	Not supported

*n* = 360. *RA* Role ambiguity, *RC* Role conflict, *RO* Role overload, *EE* Emotional exhaustion, *JS* Job satisfaction, *TI* Turnover intention.

## Discussion

This study examines the impact of role stress (RA, RC, RO) on turnover intention and determines the role of emotional exhaustion and job satisfaction in the role stress turnover intention relationship among engineers working in an Indian construction organization. The study's findings confirm the significant positive relationship between role conflict and engineers' turnover intention and the mediation of emotional exhaustion in the relationship between role conflict and engineers' turnover intention, Which is coherent with the findings of^7,11,13^. These findings imply that when an employee faces incompatible work demands from various parties that cannot be satisfied simultaneously, role conflict occurs, significantly increasing their intention to quit. Construction projects are complex, and it involves multiple parties and various trades. To effectively complete the construction project, engineers must work with two or more groups that operate quite differently. When engineers receive incompatible requests from two or more people simultaneously, it will lead to role conflict. Besides, Engineers are expected to fulfill the expectation of their job as well as the expectation of the project stakeholders; when these expectations do not align, it will result in mistakes and confusion, causing emotional exhaustion, which subsequently increases engineers' turnover intention^7^.

Contrary to the expectations, the study could not find any significant direct relationship between role ambiguity and turnover; however, the mediation effect of emotional exhaustion and job satisfaction in the role ambiguity turnover intention linkage is confirmed. These findings are partially in line with some previous results^7,13,56^. These findings suggest that when the employees perceive a lack of clarity in job expectations, it does not directly influence employee turnover; however, it indirectly affects employees' decision to quit through increased emotional exhaustion and decreased job satisfaction. An increase in role ambiguity increases employees' emotional exhaustion, which in turn increases their decision to leave. Likewise, the increase in role ambiguity decreases employees' overall satisfaction with their job, which subsequently increases their turnover intention. The respondents of this study were mainly engineers working on Indian construction projects. Construction projects are dynamic and complex, and the role expectation of construction jobs are not monotonous and varies based on the project demand. Hence the engineers working in them often face role ambiguity. This lack of clarity in construction jobs does not directly cause employee turnover; however, when the employee faces energy depletion or exhaustion due to role stress or a decrease in job satisfaction due to role stress, it will lead to an increase in intention to quit.

Similarly, the result of this study does not confirm the direct relationship between role overload and turnover intention. This insignificant relationship is in line with some of the previous studies^7,56^. However, role overload influences employees' decision to leave when mediated through emotional exhaustion. At the same time, the mediating effect of job satisfaction in the role overload turnover intention linkage is not confirmed. It is inferred from the above results that role overload does not directly influence engineers' turnover intention; however, it indirectly affects engineers' turnover intention when mediated by emotional exhaustion. Due to rapid changes in the construction sector and the variation in workforce need for construction projects, engineers are often subjected to work overload to meet project deadlines, which is very common in the construction sector. Engineers in the construction industry are generally aware of this moderately high workload involved in construction projects; hence they do not consider it an immediate threat. Therefore, an increase in role overload does not directly increase their decision to quit. However, when there is an increase in emotional exhaustion due to role overload, it significantly increases their turnover intention^7^.

This study also confirms the significant positive direct relationship between emotional exhaustion and turnover intention. This finding is in line with some previous construction studies^12,35,36^. Engineers working in construction projects face several work demands due to the dynamic nature of construction jobs; when they perceive an increase in energy depletion or exhaustion due to these demands, their attachment to their jobs decreases, increasing their turnover intention. Besides, this study's findings also confirm the negative direct relationship between engineers' overall job satisfaction and their intention to leave. These findings suggest that when engineers' satisfaction with their job decreases due to their role demands, there will be an increase in their intention to quit^7^.

Overall, the findings indicate emotional exhaustion as one of the most important predictors of engineers' intention to leave. When role stress among engineers surpasses the tolerable limit, it causes emotional exhaustion, which leads to increased withdrawal behavior or turnover intention. These findings confirm the postulates of the conservation of resources theory, which states that when employees encounter resource depletion (exhaustion) because of role stress, they resign as a coping mechanism to avoid additional resource loss^34^.

### Practical implications

One of the most critical issues that affect the construction industry, particularly in India, is the turnover of skilled professionals^14^. High employee attrition has been identified as a critical risk to the construction sector's growth and development. Hence it is essential to mitigate employee turnover in the Indian construction sector to reduce the time and cost loss associated with voluntary employee turnover. The findings of our study provide some practical implications for firms operating in the construction sector to improve employee retention. Role conflict is found to influence engineers' intention to quit directly, whereas the role ambiguity and role overload components of role stress indirectly affect engineers' turnover. Hence to mitigate employee turnover, it is essential to eliminate role stress in engineers' jobs.

Role stress in construction jobs can be reduced by implementing a clear line of command, proper information communication, and effective job design^13,24^. When there is proper communication of information and a clean line of command, engineers can clearly understand their role expectations which helps them effectively complete their work tasks. Likewise, designing construction jobs with better descriptions will assist employees in better understanding their role expectations^7^. To reduce role conflict, organizations should encourage open communication and try to avoid placing conflicting demands on engineers. Besides, the construction managers should clarify engineers' tasks while assigning a job and strive to prevent additional work to prevent work overload^7,19^.

Likewise, emotional exhaustion of engineers that occurs due to role stress is found to have a direct positive association with engineers' intention to quit. Hence, educating engineers with self-regulation strategies to monitor and regulate emotional exhaustion is essential. Appropriate training and counseling programs must be provided to engineers to handle stress and overcome emotional exhaustion. Engineers must also be supplied with adequate workplace resources and a supportive work environment to overcome emotional exhaustion^57^. Besides, HR managers must be trained to listen to workers' emotional concerns and needs regarding their jobs^13^. Similarly, the study's findings indicate a negative relation between engineers' job satisfaction and their intention to leave. Hence, construction firms should implement fair and effective human resource practices to improve engineers' job satisfaction, reducing their turnover intention^3,41^.

Specifically, the findings of this study suggest that emotional exhaustion due to role stress is a critical cause of engineers’ turnover in the Indian construction sector. Previous studies also indicate the prevalence of role stress among professionals in India^15^. Engineers working on Indian construction projects are subjected to excessive workloads and job-related complexities because of increased infrastructure operations and growing work demands, which causes exhaustion among engineers and leads to unfavorable work outcomes^58^. So, it is crucial for human resource professionals working in Indian construction firms to develop and apply stress management strategies for increasing engineers' retention. Effective stress management not only minimizes turnover but also increases construction organizations’ productivity, profit, and growth.

### Limitations and future research

This study is based only on engineers working in private construction firms across India; hence, the results cannot be generalized to other workgroups such as laborers. Further studies, including other workforce categories, are essential to generalize the findings for the construction industry as a whole. Likewise, the study is conducted only in southern India; studies on other parts of the country with a larger sample provide more robust results. Mediators other than burnout and job satisfaction, such as work-life conflict, commitment, and organizational embeddedness, can also be explored.

## Conclusions

This paper helps understand the antecedents of employee turnover in the construction sector by examining the impact of role stress on engineers' turnover intention and the mediating effect of emotional exhaustion and job satisfaction in the role stress turnover intention relationship. Prior studies have examined the impact of role stress on employees’ intention to quit. However, the mechanisms and processes that mediate the role stress and employees' turnover intention have been less explored in the construction sector. Hence the current study tries to fill this gap by examining the impact of role stress on engineers' turnover intention in the Indian construction sector, where studies related to employee turnover are scarce. The insights obtained from this study would help construction practitioners formulate strategies to reduce turnover intention. The results confirm the positive direct relationship between role conflict and turnover intention, the positive relationship between emotional exhaustion and turnover intention, and the negative relationship between job satisfaction and turnover intention. Emotional exhaustion is found to mediate the relationship between all the components of role stress and employee turnover intention. At the same time, the mediation effect of job satisfaction is significant only in the relationship between role ambiguity and turnover intention. Hence to reduce engineers' turnover, construction jobs must be designed appropriately to reduce role stress. Besides that, strategies that improve employees' job satisfaction and reduce emotional exhaustion must be planned and implemented to improve employee retention.

## Data Availability

The datasets generated during and/or analysed during the current study are available from the corresponding author on reasonable request.
